# Application of probiotic therapy in nonalcoholic fatty liver disease: mediating mechanism and future perspective

**DOI:** 10.3389/fcimb.2025.1638372

**Published:** 2025-09-15

**Authors:** Xing-Yu Luo, Wei-Bin Huang, Chuang-Hui Lu, Wu Gu, Zi-Xuan Feng, Sui Shen, Ming-Zheng Chen, Shu-Sen Zheng, Zhe Yang

**Affiliations:** ^1^ Department of Hepatobiliary and Pancreatic Surgery, Key Laboratory of Artificial Organs and Computational Medicine in Zhejiang Province, Shulan (Hangzhou) Hospital, Shulan International Medical College, Zhejiang Shuren University, Hangzhou, China; ^2^ School of Medicine, Wenzhou Medical University, Wenzhou, China; ^3^ Graduate School, Zhejiang Chinese Medical University, Hangzhou, China; ^4^ School of Clinical Medicine, Hangzhou Normal University, Hangzhou, China; ^5^ Department of Hepatobiliary and Pancreatic Surgery, Shulan (Boao) Hospital, Boao, China

**Keywords:** probiotic, nonalcoholic fatty liver disease, application, mediating mechanism, future perspective

## Abstract

Nonalcoholic fatty liver disease (NAFLD) has a global prevalence of 20%-33%, and has become the main cause of chronic liver disease. Apart from lifestyle modification therapy, there is currently no definitive pharmacological treatment, thus there is an urgent need to find effective intervention strategies to treat NAFLD. With the discovery of the important role of gut microbes in the pathogenesis of NAFLD, research on the prevention and treatment of non-alcoholic fatty liver disease by probiotics is increasing. At present, many studies have confirmed the role of probiotic regulation in the treatment of NAFLD, which can reduce the level of transaminase and liver fibrosis in patients and protect the liver. The clinical application of probiotics includes single species such as *Lactobacillus* and *Bifidobacteria*, as well as synbiotics with different compositions. This article reviews the therapeutic effects of probiotics on NAFLD and the mechanisms by which probiotics directly or indirectly affect the disease. Further research is needed to fully understand the specific underlying mechanisms between probiotics, gut microbes, and NAFLD, and more large-scale clinical trials are needed to evaluate probiotics for the treatment of NAFLD.

## 1 Introduction

Nonalcoholic fatty liver disease (NAFLD) refers to liver disease in which more than 5% of liver cells are infiltrated with liver fat on liver biopsy specimens and with no regard to excessive alcohol consumption or other clear liver injury factors (Roychowdhury et al., 2018). NAFLD can be divided into nonalcoholic simple fatty liver (NAFL) and nonalcoholic steatohepatitis (NASH). It is worth mentioning that since most NAFLD patients have one or more cardiometabolic risk factors, the existing name of NAFLD focuses on excluding excessive drinking as the cause. Thus, some international experts have reached a consensus and proposed using the new term metabolic dysfunction-associated fatty liver disease (MAFLD), which refers to steatosis brought on by an unbalanced metabolic environment, along with potentially serious steatohepatitis lesions and accompanying fibrosis, to replace NAFLD (Eslam et al., 2020). NAFLD and MAFLD overlap significantly, and the two classifications generally have strong concordance, as indicated by a Cohen kappa value of up to 0.92. However, current research related to MAFLD are very limited (Alboraie et al., 2019). Currently, the global prevalence of NAFLD is increasing, with the incidence rate ranging from 30% to 32.4% (Riazi et al., 2022; Brennan et al., 2023; Younossi et al., 2023). Without timely treatment, NAFLD can progress to cirrhosis, hepatocellular carcinoma, and even death (Friedman et al., 2018). However, there is no definite drug therapy for steatosis except lifestyle interventions. Therefore, it is urgent need to find effective treatment methods to alleviate NAFLD.

## 2 Application of probiotic therapy in nonalcoholic fatty liver disease

### 2.1 NAFLD and probiotic therapy

Recent studies have shown that regulation of gut microbiota can be a feasible strategy for preventing and treating NAFLD. Probiotics are live microorganisms that, when administered in adequate amounts, confer a health benefit on the host. Studies have found that probiotic therapy is an important means of regulating gut microbiota (O'flaherty and Klaenhammer, 2010). At present, many studies (Gao et al., 2016; Loman et al., 2018; Tang et al., 2019; Xiao et al., 2019; Pan et al., 2020; Yang et al., 2021; Huang et al., 2022) have confirmed the role of probiotics regulation in NAFLD treatment through meta-analysis, which can reduce the level of transaminase and liver fibrosis in patients and protect the liver (Khan et al., 2019; Sabirin et al., 2022). Therefore, the first part of the review focuses on the application of probiotic therapy in NAFLD, aiming to provide more and better ideas for the prevention and treatment of NAFLD. This review will provide an updated synthesis of the mechanisms of probiotic therapy in NAFLD and its therapeutic potential, with a focus on novel insights and future research directions.

### 2.2 The role of probiotics in NAFLD

#### 2.2.1 Single probiotic

The protective and preventive function of *Lactobacillus* in NAFLD has been fully studied. *Lactobacillus rhamnosus* GG has been confirmed to share intestinal fatty acids and prevent the development of diet-induced hepatic steatosis, thus effectively treating NAFLD (Jang et al., 2019). Ritze et al (Ritze et al., 2014). also showed that *Lactobacillus rhamnosus* GG can prevent NAFLD in mice. Mu et al (Mu et al., 2020). showed that *Lactobacillus fermentum* CQPC06 can colonize in the intestinal tract and alter gut microbiota in NAFLD mice. *Lactobacillus paracasei* CNCM I-4034 and *Lactobacillus rhamnosus* CNCM I-4036 can relieve the liver injury by reducing gene expression of pro-inflammatory macrophage cell and leukocyte infiltration of the liver in NAFLD rats (Fontana et al., 2021). Through the *in vitro* model, *Lactobacillus plantarum* AR113 and *Lactobacillus casei* pWQH01 relieved steatosis in a manner dependent on bile salt hydrolase (Huang et al., 2020). Similarly, *Lactobacillus sakei* MJM60958 can significantly inhibit lipid accumulation in HepG2 cells stimulated by oleic acid and cholesterol, reduce weight of both body and liver in NAFLD mice and control the level of NAFLD-related markers as well, indicating that *Lactobacillus sakei* MJM60958 can also effectively prevent and treat NAFLD (Nguyen et al., 2022b). *Lactobacillus acidophilus* SNZ 86 which can enrich selenium has also been confirmed to relieve hepatic steatosis by up-regulating the adenosine 5’-monophosphate (AMP)-activated protein kinase (AMPK) and silent information regulator 1 (SIRT-1) pathways (Pant et al., 2022). *Lactobacillus paracasei* Jlus66, isolated from natural fermented milk, also has great potential in preventing NAFLD (Ye et al., 2017), which was consistent with that of Wang et al (Wang et al., 2019). In addition, Geng et al (Geng et al., 2022). identified a new type of probiotic *Lactobacillus kefiranofaciens* ZW3 through zebrafish model and explored the its effect on lipid deposition. They proved that *Lactobacillus kefiranofaciens* ZW3 has a specific protective effect on NAFLD. Interestingly, engineering *Lactobacillus reuteri*, made by Oh et al., exerted the further therapeutic effect in NAFLD through recombinant Interleukin-22 (IL-22) delivery (Oh et al., 2020). Liu et al (Liu et al., 2022). made lactoferrin expressed by recombinant lactic acid bacteria, which was more effective in relieving steatosis.

*Bifidobacteria* also play an important role in the protection and prevention of NAFLD disease. Yan et al (Yan et al., 2020). evaluated the effect of *Bifidobacterium lactis* V9 on hepatic steatosis in NAFLD rats induced by high-fat diet. They found that *Bifidobacterium lactis* V9 could inhibit inflammation and relieve NAFLD. Do et al (Do et al., 2022). also found *Bifidobacterium animalis ssp. lactis* MG741 could reduce weight and relieve NAFLD by relieving intestinal permeability and inflammatory cytokines. Oral *Bifidobacterium longum* supplements can prevent obesity and NAFLD by regulating the mRNA expression of renin-angiotensin system components (MaChado et al., 2021). *Bifidobacterium longum* and *Lactobacillus acidophilus* can reduce liver fat accumulation, with the former being more effective (Xu et al., 2011).

Moreover, the therapeutic potential of other strains in NAFLD disease can’t be ignored. *Faecalibacterium prausnitzii* LC49 and LB8 were able to produce short-chain fatty acid and regulate the gut microbiota, indicating their potential role in NAFLD (Hu et al., 2022). MIYAIRI 588, as a probiotic that can enhance butyrate production, has been discovered to slow down the progression of NAFLD (Endo et al., 2013). Seo et al (Seo et al., 2013). also showed that MIYAIRI 588 had new potential to relieve NAFLD. In addition, *Limosilactobacillus fermentum* MG4295 has been proved to relieve hyperglycemia, a complication of NAFLD (Kim et al., 2022). (**Table 1**).

**Table 1:** Single probiotic.

Author	Category	Probiotics	Functions	Reference
Jang et al. Ritze et al.	Lactobacillus	Lactobacillus rhamnnosus GG	Share intestinal fatty acids and prevent the hepatic steatosis	(Ritze et al., 2014; Jang et al., 2019)
Mu et al.	Lactobacillus	Lactobacillus femenhim COPC06	Colonize in the intestinal tract and alter gut microbiota	(Mu et al., 2020)
Fontana et al.	Lactobacillus	Lactobacillus paracasei CNCM1-4034, Lactobacillus rhamnosus CNCM I-4036	Reduce gene expression of pro-inflammatory macrophage cell and leukocyte infiltration and relieve the liver injury	(Fontana et al., 2021)
Huang et al.	Lactobacillus	Lactobacillus plantarum AR113, Lactobacillus casei pWOH01	Relieve steatosis in a manner dependent on bile salt hydrolase	(Huang et al., 2020)
Nguyen et al.	Lactobacillus	Lactobacillus sakei MIM60958	Inhibit lipid accumulation in HepG2 cells	(Nguyen et al., 2022b)
Pant et al.	Lactobacillus	Lactobacillus acidophilus SNZ 86	Upregulate the AMPK and SIRT-I pathways	(Pant et al., 2022)
Ye et al. Wang et al.	Lactobacillus	Lactobacillus paracasei Jhs66	Have great potential in preventing NAFLD	(Ye et al., 2017; Wang et al., 2019)
Geng et al. Liu et al.	Lactobacillus	Lactobacillus kefiranofaciens ZW3, Lacticacid bacteria	Effectively relieve steatosis	(Geng et al., 2022; Liu et al., 2022)
Oh et al.	Lactobacillus	Engincering Lactobacillus reuteri	Treat NAFLD further through recombinant Interleukin-22 (IL-22) delivery	(Oh et al., 2020)
Yan et al.	Bifidobacteria	Bifidobacterium lactis V9	Inhibit inflammation and relieve NAFLD	(Yan et al., 2020)
Do et al.	Bifidobacteria	Bifidobacterium animalis ssp. lactis MG741	Relieve intestinal permeability and inflammatory cytokines	(Do et al., 2022)
Machado et al.	Bifidobacteria	Oral Bifidobacterium longum supplements	Regulate the mRNA expression of renin-angiotensin system components	(MaChado et al., 2021)
Xu et al.	Bifidobacteria	Bifidobacterium longum and Lactobacillus acidophilus	Reduce liver fat accumulation	(Xu et al., 2011)
Hu et al.	Other strains	Faecalibacterium prausnitzii LC49 and LB8	Produce short-chain fatty acid and regulate the gut microbiota	(Hu et al., 2022)
Endo et al Seo et al.	Other strains	MIYAIRI 588	Slow down the progression of NAFLD	(Endo et al., 2013; Seo et al., 2013)
Kim et al.	Other strains	Limosilactobacillus fermentum MG4295	Relieve hyperglycemia	(Kim et al., 2022)

#### 2.2.2 The combination of multi-strain probiotics

The use of single probiotics may not be satisfactory for the treatment of NAFLD. Therefore, many basic studies have focused on the research of the role of the combination of two or more probiotics. Yu et al (Yu et al., 2021). found that *Lactobacillus lactis* and *Pediococcus pentosaceus* could significantly postpone the progress of NAFLD through the intestinal-liver axis, especially through the tryptophan metabolic pathway. In the rat NAFLD model, Azarang et al (Azarang et al., 2020). showed that the utilization of single probiotics such as *Lactobacillus acidophilus*, *Lactobacillus casei, Lactobacillus reuteri* and *Bacillus coagulans* could reduce oxidative stress markers and the combination of those four probiotics could significantly relieve more symptoms of NAFLD. Regular use of compound probiotics “Symbiter” has also been confirmed to prevent monosodium glutamate-induced NAFLD in mice (Savcheniuk et al., 2014). Furthermore, a probiotic blend containing five different Bacillus genera has been shown to effectively reverse high-fat diet-induced hepatic steatosis, highlighting the potential of Bacillus in treating NAFLD (Kim et al., 2018). Mutaflor^®^ probiotics have also been shown to slow the progress of NAFLD by regulating HSC signaling (Hany et al., 2022).

Clinical trials investigating the role of combined probiotics in NAFLD have been successfully conducted, further enhancing the potential of probiotic combinations for clinical treatment of NAFLD. Tablets containing *Lactobacillus bulgaricus* and *Streptococcus thermophilus* was reported to be able to improve the level of liver transaminase in patients with NAFLD, thus having a better therapeutic effect on NAFLD (Aller et al., 2011). In a study conducted by Kobyliak et al (Kobyliak et al., 2018c), 58 patients with type 2 diabetes and NAFLD were enrolled and randomly assigned to receive either the polyprobiotic “Symbiter” or a placebo. The researchers discovered a significant reduction in the fatty liver index, accompanied by decreased serum levels of aspartate aminotransferase (AST), γ-glutamyl transpeptadase (GGT), tumor necrosis fator (TNF), and interleukin-6 (IL-6) in the probiotic group. These observations indicated the potential of “Symbiter” probiotics as a treatment for NAFLD.

Ahn et al (Ahn et al., 2019). treated obese NAFLD patients with a mixture of probiotics including six bacteria. They found that probiotic treatment for 12 weeks significantly reduced intrahepatic fat and body weight in NAFLD patients. Probiotic capsules composed of *Bifidobacterium longum* and *Lactobacillus acidophilus* have been confirmed to significantly reduce body weight, body mass index, waist and hip circumference and TNF-α levels in patients with NAFLD, and increase the level of serum total antioxidant capacity (Javadi et al., 2018). Similarly, Lactocare, a probiotic capsule containing seven beneficial strains, significantly reduced blood glucose and inflammatory markers in patients with NAFLD (Sepideh et al., 2016). Multi-strain probiotics (MCP^®^ BCMC^®^ strains) containing six different lactic acid bacteria and bifidobacteria complement the treatment of NAFLD can stabilize mucosal immune function and protect NAFLD patients with increased intestinal permeability (Mohamad Nor et al., 2021). VSL#3, a probiotic blend containing eight cultured bacteria, has been utilized in the treatment of NAFLD in rats via its therapeutic effects involve the mitigation of oxidative stress and alleviation of inflammatory liver injury (Esposito et al., 2009). Derosa et al (Derosa et al., 2022). recruited 60 white adult suffering from NAFLD who were randomly assigned to receive VSL#3 or placebo. The results showed that VSL#3 probiotic therapy could significantly improve liver parameters and ultrasonic grading, and there was no difference between men and women. Loguercio et al (Loguercio et al., 2005). also found that probiotic VSL#3 can significantly improve liver injury caused by NAFLD through clinical cohort study. The capsule formed by probiotic combination has also been fully explored in pediatric NAFLD. Compared with children who received placebo, the level of liver function of children who received probiotic capsules exhibited remarkable enhancement (Famouri et al., 2017) (**Table 2**).

**Table 2:** The combination of probiotics.

Authors	The combination of Probiotics	Functions	Reference
Yu et al.	Lactobacillus lactis, Pediococcus pentosaceus	Significantly postpone the progress of NAFLD through the intestinal-liver axis	(Yu et al., 2021)
Azarang et al.	Lactobacillus acidophilus, Lactobacillus casei, Lactobacillus reuteri, Bacillus coagulans	Significantly relieve more symptoms of NAFID	(Azarang et al., 2020)
Savcheniuk et al. Kobyliak et al.	“Symbiter” (containing 14 probiotics)	Decrease serum levels of AST, GGT, TNF and IL-6 and prevent monosodium glutamate-induced NAFLD	(Savcheniuk et al., 2014; Kobyliak et al., 2018c)
Kim et al.	A probiotic blend containing five different Bacillus genera	Effectively reverse high-fat diet-induced hepatic steatosis	(Kim et al., 2018)
Hany et al.	Mutaflor^®^ probiotics	Slow the progress of NAFLD by regulating HSC signaling	(Hany et al., 2022)
Aller et al.	Lactobacillus bulgaricus, Streptococcus thermophilus	Improve the level of liver transaminase in patients with NAFLD	(Aller et al., 2011)
Ahn et al.	Lactobacillus acidophilus, L.rhamnosus, L.paracasei Pediococcus pentosaceus, Bifdobacterium lactis, B. breve	Reduce intrahepatic fat and bodyweight in NAFLD patients	(Ahn et al., 2019)
Javadi et al.	Bifidobacterium longum, Lactobacillus acidophilus	Reduce body weight, body mass index, waist and hip circumference and TNF-α levels and increase the level of serum total antioxidant capacity	(Javadi et al., 2018)
Sepideh et al.	Lactocare (containing seven beneficial strains)	Reduce blood glucose and inflammatory markers in patients with NAFLD	(Sepideh et al., 2016)
Mohamad et al.	MCP^®^ BCMC^®^ strains (containing six different lactic acid bacteria and Bifidobacteria)	Stabilize mucosal immune function and protect NAFLD patients with increased intestinal permeability	(Mohamad Nor et al., 2021)
Esposito et al. Derosa et al. Loguercio et al.	VSL#3 (a probiotic blend containing eight cultured bacteria)	Mitigate oxidative stress and alleviate inflammatory liver injury, improve liver parameters and ultrasonic grading, effectively relieve liver injury	(Loguercio et al., 2005; Esposito et al., 2009; Derosa et al., 2022)

#### 2.2.3 Incorporation of probiotics with other biological components

Since individual probiotic and combination of probiotics display have shown promising therapeutic potential, the incorporation of probiotics with other biological components has also attracted wide attention. Ahmed et al (Ahmed et al., 2020). showed that the combination of *Lactobacillus reuteri* and metronidazole could effectively regulate intestinal flora of NASH mice, resulting in improved therapeutic outcomes. Wang et al (Wang W. et al., 2020). found that the combination of probiotics *Bifidobacterium bifidum* V, *Lactobacillus plantarum* X and *Salvia miltiorrhiza* polysaccharide effectively alleviates hepatic steatosis by modulating gut microbiota and relieving insulin resistance in high-fat diet induced NAFLD mice. Importantly, the combined treatment showed potential benefits surpassing those of probiotics *Bifidobacterium bifidum* V and *Lactobacillus plantarum* X alone, indicating that *Salvia miltiorrhiza* polysaccharide can enhance the function of these probiotics. As the substrate of prebiotics, when combined with *Bifidobacteria*, it has the potential to improve the efficacy of NAFLD treatment. It is confirmed that the combination of Resveratrol and *Bifidobacteria* may be a potential drug for the treatment of NAFLD (Hu et al., 2021). In addition, in the NAFLD rat model, “Symbiter” combined with Omega-3 therapy could significantly relieve liver steatosis and liver conversion lipid accumulation compared with probiotics alone (Kobyliak et al., 2017). Furthermore, Kobyliak et al (Kobyliak et al., 2018a). incorporated 48 patients with type 2 diabetes mellitus complicated with NAFLD and randomly assigned them to multi-strain “Symbiter” combined with Omega-3 (“Symbiter Omega” combination) and placebo respectively. They found that “Symbiter Omega” combination could reduce liver fat, improve blood lipids and metabolic characteristics, and reduce chronic systemic inflammation in NAFLD patients. Smectite is a natural silicate that binds to digestive mucus and has the ability to bind endotoxin and exotoxin. Studies have found that the combination of multi-probiotics “Symbiter” and Smectite gel “Symbiter Forte” can play a synergistically enhanced role in the effective treatment of NAFLD (Kobyliak et al., 2018b). In a clinical trial, 80 patients with NAFLD were given symbiotic supplements (including six probiotics and fructooligosaccharides) and placebos respectively. Symbiotic supplements have been found to relieve steatosis in patients with NAFLD (Asgharian et al., 2016). Probiotic mixtures have been found to act on lipid profiles, leptin and inflammatory biomarkers to treat fatty liver disease (Al-Muzafar and Amin, 2017). Similarly, Crommen et al (Crommen et al., 2022). have shown in clinical trials that a mixture of multi-strain probiotic powder and specific trace microelements can effectively improve NAFLD-related markers in obese patients undergoing miniature gastric bypass surgery.

#### 2.2.4 Probiotics related products

The possible impact of probiotics-related products on NAFLD has garnered significant attention. Kefir is a probiotic beverage that contains a variety of lactic acid bacteria and yeast. In the NAFLD mouse model, the administration of Kefir has been shown to regulate the composition of intestinal microbiota and fungal flora, leading to effective treatment of the condition (Kim et al., 2017). Kombucha is a kind of natural nonalcoholic fermented beverage with probiotic characteristics produced by symbiotic culture of bacteria and yeast. Moreira et al (Moreira et al., 2022). successfully confirmed that Kombucha can improve glucose tolerance and reduce liver steatosis in obese mice through NAFLD mouse experiments. Moreover, Konda et al (Konda et al., 2020). found that probiotics banana juice treated by pectinase can effectively deal with liver steatosis to effectively prevent NASH.

#### 2.2.5 Probiotics plus lifestyle intervention

It is worth mentioning that probiotic supplements in conjunction with lifestyle interventions have also been confirmed to have positive effects on blood glucose parameters and leptin levels in patients with NAFLD (Behrouz et al., 2017). Lifestyle changes with multi-strain probiotic therapy can significantly improve liver histology, the levels of alanine aminotransferase and cytokine in patients with NAFLD (Duseja et al., 2019). Exercise training and probiotics are also recommended as effective treatments for NAFLD. Hosseini et al (Hosseini et al., 2022). proved that intensive interval training and *Lactobacillus rhamnosus* GG can minimize damage to liver tissue cell and inflammation caused by NAFLD.

### 2.3 Future expectations

The aforementioned studies have consistently demonstrated the efficacy of probiotics and their associated products in the prevention and treatment of NAFLD. Furthermore, there is a growing trend in research towards the clinical application of these practical products. The application prospect of probiotics and its related products in NAFLD is worth anticipation and further promoting.

The mechanism underlying the therapeutic effects of probiotics in NAFLD treatment has consistently been a focal point of research. It is believed that distinct probiotic strains may exert their effects through different mediating mechanisms. Utilizing probiotics allows researchers to observe changes in the individual’s gut microbiota composition, while investigating how these changes impact disease progression remains a key area for exploration. With the continuous advancement of technical tools, an increasing number of research methods have been employed to investigate the mechanism underlying probiotic treatment of NAFLD. However, the current understanding of the precise mechanism by which probiotics exert their effects in NAFLD treatment remains limited. Only through the comprehensive utilization of various research techniques can a more comprehensive understanding of the mediating mechanisms be achieved. Researchers must devote further efforts to clarify the specific mechanism through which different probiotics play a role in NAFLD.

## 3 The mediating mechanism of probiotics in the treatment of nonalcoholic fatty liver disease

Probiotic regulation offers an effective strategy for the treatment and prevention of NAFLD, particularly in the absence of clear pharmacological interventions for steatosis. Understanding the mediating mechanisms underlying probiotic therapy in NAFLD has remained a central focus of research focus of research. Probiotics have the ability to modulate the physiological function and metabolic status of patients with NAFLD by influencing the composition, abundance and balance of intestinal microflora. To investigate the mediating mechanism of probiotic therapy, it is essential to commence with a comprehensive exploration of the common pathogenesis and etiology of NAFLD.

The ecological imbalance of intestinal flora, alterations of intestinal cell permeability, liver injury, endoplasmic reticulum stress, abnormal activation of cellular signaling pathway, as well as dietary and genetic factors of patients, have all been implicated in the occurrence of NAFLD (Williams et al.,; Mouzaki and Allard, 2012; Goodwin et al., 2013). The second part of the review aims to comprehensively explore the intricate mediating mechanisms of probiotics in NAFLD treatment, contributing to the development of novel therapeutic approaches for the disease.

### 3.1 Maintaining the integrity of intestinal epithelial cells: anti-oxidation and anti-inflammation

#### 3.1.1 Reactive oxygen species and Intestinal inflammation

Intestinal inflammation can influence the intestinal-liver axis, damaging the intestinal barrier, leading to bacterial translocation, activating the immune system response, and triggering a series of pro-inflammatory pathways in the liver, thereby accelerating the process of NAFLD (Pierantonelli and Svegliati-Baroni, 2019).

ROS in human body is mainly produced in endoplasmic reticulum, peroxisome, mitochondria and other organelles. Specially, Reactive oxygen species (ROS) production mainly occurs during the mitochondrial electron transport chain process (Novak and Mollen, 2015). However, excessive ROS can impede electron transfer, leading to mitochondrial damage and disruption of biological function of mitochondria and cell homeostasis, ultimately causing cell death (Kiffin et al., 2006; Scherz-Shouval and Elazar, 2007; Novak and Mollen, 2015).

The excessive accumulation of ROS in cells result in oxidative stress, characterized by an imbalance between ROS production and clearance in cells and tissues. In response to oxidative stress, cells activate various defense mechanisms or undergo cell death. Oxidative stress can induce intestinal mucosal damage, increase intestinal epithelial barrier permeability, facilitate bacterial invasion, stimulate immune response and initiate the pathological process of intestinal inflammation. The key manifestations of active intestinal inflammation include immune cell infiltration and neutrophilic granulocytosis (Goyette et al., 2007).

#### 3.1.2 Probiotics prevent and treat NAFLD by preventing intestinal inflammation and antioxidation

##### 3.1.2.1 Genetic engineering *Escherichia coli*

*Escherichia coli* Nissle 1917 (ECN) is a genetically engineered oral probiotics with good safety and can assist in the treatment of many kinds of diseases (Lynch et al., 2022; Zhou et al., 2022). ECN-pE, an oral probiotic, was genetically modified to enhance the expression of catalase and superoxide dismutase (SOD) for the treatment of intestinal inflammation. ZhouJ et al. evaluated the SOD competence of different ECN subtypes by assessing their ability to scavenge superoxide. Notably, ECN-pE(C/A)2 exhibited strong SOD activity, promoting significant colon tissue repair and alleviating intestinal inflammation (Zhou et al., 2022).

##### 3.1.2.2 Bifidobacterium longum

*Bifidobacterium longum* have been demonstrated their ability to inhibit the development of intestinal inflammation by regulating immune system balance, enhancing acetate production and improving intestinal mucosal barrier function (Underwood et al., 2015; Chichlowski et al., 2020; Yao et al., 2021). In a study conducted by F.A.Abrantes et al., it was shown that *Bifidobacterium longum B.longum5^1A^* effectively reverse colitis-induced increase of intestinal permeability and reduce the degree of colonic lesions (Abrantes et al., 2020). Its molecular biological mechanism is alleviating a series of changes during intestinal inflammation, such as decreased eosinophil peroxidase level, increase of IL-1β level, the significant increase of immunoglobulin concentration and the increase of inflammatory markers (Dvorak et al., 1994; Abrantes et al., 2020).

S. Yan et al. confirmed that the metabolites of *B.longum* YS108R contain antioxidant substances (Yao et al., 2021). Yusheng Wang et al. demonstrated that the supernatant from cultured *B.longum* CCFM752 exhibits antioxidant effect on cells, enhancing catalase activity and decreasing NADPH oxidase activity. In addition, it has been proved that *Lactobacillus sake* and other *Lactobacillus* probiotics can also relieve the symptoms of NAFLD through antioxidant mechanism (Wang et al., 2021).

Genetic engineering *Escherichia coli* and *Bifidobacterium longum* can improve the antioxidant level of cells and tissues, mitigate ROS-induced cell damage, protect intestinal mucosal barrier and effectively inhibit NAFLD triggered by intestinal inflammation. These findings highlight their significant clinical application value.

### 3.2 Regulating lipid metabolism to relieve NAFLD

#### 3.2.1 Core pathological process

The main cause of NAFLD is the excessive accumulation of fat in the liver (Kanuri and Bergheim, 2013; Zeigerer, 2021). The fat accumulated in the liver can originate from various sources, including fatty acids digested and absorbed by intestinal epithelial cells from food, *de novo* synthesis of body fat, adipose tissue fat transport and conversion of other substances (Jones, 2016; Alves-Bezerra and Cohen, 2017; Carotti et al., 2020; Badmus et al., 2022). The liver, as the central organ of fatty acid metabolism, will experience fat accumulation when the production of fatty acids exceeds their consumption, including fat transport (Alves-Bezerra and Cohen, 2017). In the existing scientific research, it has been found that some probiotics can alleviate NAFLD symptoms by modulating lipid metabolism in the liver, offering potential avenues for clinical treatment of NAFLD.

#### 3.2.2 Mechanism of *Lactobacillus sake* regulating lipid metabolism in liver

*Lactobacillus sak*e has good antibacterial and antioxidant effects, exhibiting excellent antibacterial efficacy, safety and tolerance in the body (Schillinger and Lücke, 1989; Amanatidou et al., 2001; DÜz et al., 2020). Huong Thi Nguyen et al. evaluated the high anti-lipid effect of *Lactobacillus sake* MJM60958 in HepG2 cells and its therapeutic effect for high-fat diet induced NAFLD mouse model. Among different strains, MJM60958 showed the most pronounced effect in inhibiting lipid synthesis. It reduced lipid accumulation in hepatocytes, relieving NAFLD by decreasing serum level of AST, ALT, Triglyceride (TG) and total cholesterol (TCHO), which serve as key markers of NAFLD (Nguyen et al., 2022a).

#### 3.2.3 Mechanism of *Lactobacillus salivarius* regulating lipid metabolism in liver

Peroxisome proliferator activated receptors (PPAR) α, β/δ and γ regulate lipid homeostasis in the liver. Among them, PPARα is a key nuclear receptor, which controls the oxidation rate of fatty acids in mitochondria and is also related to carnitine palmitoyltransferase-1. Specifically, PPARα controls the oxidation of fatty acids in mitochondria, and PPARγ is involved in adipogenesis and lipid storage. Additionally, the AMPK pathway, which is activated in response to metabolic stress, plays a significant role in regulating lipid metabolism (Wang Y. et al., 2020).

In the study of Lihui Zhu et al., the probiotic strain *Lactobacillus salivarius* SNK-6 (*L.salivarius* SNK-6) demonstrated beneficial effects in a lying hen model of NAFLD. The findings revealed that the inhibition of miR-130a-5p significantly increased the expression of PPAR α, PPAR γ, fatty acid binding protein 4 (FABP4), SREBP1 and fatty acid synthase (FASN) related genes. Conversely, the administration of *L.salivarius* SNK6 up-regulate the expression of miR-130a-5p and down-regulate the expression of MBOAT2. Through the miR-130a-5p/MBOAT2 pathway, *Lactobacillus salivary* SNK-6 reduced the activity of ALT and AST and inhibited hepatic fat deposition, thus relieving the condition of NAFLD (Zhu et al., 2022).

#### 3.2.4 Mechanism of *Lactobacillus plantarum* regulating lipid metabolism in liver

Studies conducted by Chuan Li et al. have shown that *L.plantarum* NCU116 alleviate hepatic fat accumulation by downregulating fat production and upregulating the expression of genes associated with fat decomposition and fatty acid oxidation. The experimental group treated with *L.plantarum* NCU116 showed increased expression of PPAR α, PPAR γ, PPAR δ, PGC1 α and CPT1 α, leading to effective reduction of hepatic fat accumulation (Li et al., 2014).

#### 3.2.5 Mechanism of *Lactobacillus reuteri* regulating lipid metabolism in liver

Carmen Tenorio-Jim é nez et al. conducted the clinical evaluation trial of *Lactobacillus reuteri* V3401 on NAFLD. Sixty participants (aged 18 to 65 years) diagnosed with IRS were randomized in a 1:1 ratio to receive either a daily dose of placebo or 5×10^9^ colony-forming units of L. reuteri V3401. The study aimed to explore the mediating mechanism of *L. reuteri* in relieving NAFLD by detecting human serum level of LPS, insulin resistance and liver steatosis after the application of *L. reuteri*. Currently, the study is still in the stage of experiment and data analysis (Tenorio-Jiménez et al., 2018).

### 3.3 Probiotics relieve NAFLD by regulating the levels of different cytokines

#### 3.3.1 Effect of *Bifidobacterium* on the level of related cytokines

Tumor necrosis factor-α (TNF-α), interleukin 1 beta (IL-1β) and interleukin-18 (IL-18) are the key cytokines in the pathogenesis of NAFLD (Stojsavljević et al., 2014; Ezquerro et al., 2019).

Experiments evidence has demonstrated that *Lactobacillus* sake MJM60958 relieved NAFLD by reducing the level of TNF-α (Carotti et al., 2020). In a Study by Moon Ho Do et al., mice fed with high-fat diet exhibited increased expression of genes encoding inflammatory cytokines TNF-α, IL-1β and IL-6. However, mice treated with a high dose of *Bifidobacterium* lactose MG741 reversed the expression trend of these genes, improving intestinal permeability and offering potential therapeutic benefits for NAFLD (Do et al., 2022).

#### 3.3.2 Effect of multiple probiotics combination on the level of related cytokines

After clinical intervention with multiprobiotic “Symbiter” in patients with NAFLD, the levels of AST and GGT which are related with fat synthesis and the levels of TNF-α, IL-1β, IL-6, IL-8 and INF-γ decreased significantly. The fatty liver index (FLI) was significantly improved (Kobyliak et al., 2018a; Kobyliak et al., 2018c).

#### 3.3.3 The function of IL-17

The regulation of cytokines and improvement of the tissue microenvironment are important for treating liver inflammation and alleviating autoimmune diseases. Interleukin-17 (IL-17) has shown promising potential in both research and application (Zhang et al., 2015). IL-17 serves as a key initiator of the inflammatory response, promoting the release of inflammatory cytokines and inducing an inflammatory cascade. Upon binding to its receptor, IL-17 can play its biological role through mitogen-activated protein kinase (MAPK) pathway and activating transcription factors such as activator protein-1 (AP1), CCAAT-enhancer-binding proteins (C/EBPs) and nuclear transcription factor κB (NF-κB) (Monin and Gaffen, 2018).

The clinical studies of Chung-Hsing Wang et al. showed that the serum levels of IL-8, IL-17, MIP-1β and TNF-α in patients with type I diabetes treated with *Bifidobacterium animalis*, *Akkermansia muciniphil*a and *Lactobacillus salivarius* were significantly lower than those without probiotics (Wang et al., 2022).

However, experimental study by Shuying He et al. has shown that IL-17 from Th17 cells can restore the function of intestinal epithelial tissue and barrier and maintain the integrity of intestinal barrier (He et al., 2022). To some extent, this finding highlighted the dual regulatory effect of IL-17 varing depending on the specific circumstances.

Currently, there is a scarcity of animal experiments and clinical trials investigating the correlation between IL-17 level changes and NAFLD improvement following probiotic use. Further research is needed to clucidate the relationship between IL-17 levels and NAFLD.

#### 3.3.4 Blueberry combined with probiotics to regulate the level of cytokines

##### 3.3.4.1 Effects of bioactive substances from blueberry on inflammation and oxidative stress

According to Felgus-Lavefve L et al., the bioactive molecules of blueberry inhibited inflammation and oxidative stress by downregulating NF-κB pathway, reducing ROS levels and attenuating lipid peroxidation. The understanding of the main molecular mechanisms of blueberry chemicals in the cell model is progressively advancing (Felgus-Lavefve et al., 2022).

Tarfa Albrahim et al. showed that the contents of antioxidant enzymes, glutathione and lipid peroxidation in rats fed blueberries increased, while the activities of inflammatory mediators (TNF-α, IL-6 and nuclear factor kappa light chain enhancer of activated B cells) and fibrosis marker transforming growth factor β1 (TGF-β1) in rat liver decreased significantly (Albrahim and Alonazi, 2022).

The studies above demonstrate the significant role of blueberry’s biological activity in anti-oxidation and anti-inflammation, leading to a notable reduction of inflammatory mediators-related cytokines. When blueberry is used in combination with probiotics, it exhibits distinct effects on the level of cytokines in NAFLD model.

##### 3.3.4.2 Blueberry in combination with probiotics & important cytokine IL-22 and its molecular pathway

Studies by Juanjuan Zhu et al. have shown that blueberry combined with probiotics can alleviate NAFLD through IL-22-mediated Janus kinase 1 (JAK1)/signal transducer and activator of transcription 3 (STAT3)/Bcl-2-associated X protein (BAX) signal pathway (Zhu et al., 2018).

The expression of IL-22, JAK1 and STAT3 in NAFLD model significantly decreased, while the expression of apoptosis factor BAX showed a marked increase. However, the administration of probiotics resulted in a substantial increase in the levels of IL-22, JAK1 and STAT3 in NAFLD model, while decreased the level of BAX. Similarly, the suppression of IL-22 hindered the ability of probiotics to promote the expression of JAK1, STAT3 and BAX (Zhu et al., 2018). Probiotics can activate the JAK1/STAT3 signal pathway, inhibit the apoptosis factor BAX and reduce lipid deposition *in vitro* through IL-22 (Zenewicz, 2018; Zhu et al., 2018).

In addition, IL-22, acts as a key regulator of epithelial homeostasis, playing a critical role in preserving the function of epithelial barrier (Zenewicz, 2018; Patnaude et al., 2021).

##### 3.3.4.3 Microflora secreting IL-22

IL-22 secreted by engineered *Lactobacillus reuteri* significantly reduced liver weight and triglyceride content in NAFLD model (Oh et al., 2020). It can be seen that IL-22 can alleviate the development of NAFLD.

IL-22, a member of the IL-20 subfamily, controls lipid metabolism in the liver by activating the above signaling pathways (Pan et al., 2014). As a therapeutic protein, IL-22 holds promising prospects for mitigating nonalcoholic fatty liver disease.

### 3.4 Probiotics relieve NAFLD by regulating intestinal microflora

#### 3.4.1 Intestinal microbiota and imbalance

Intestinal microbiota encompasses the diverse microbial community residing in the human intestinal tract. These microorganisms are involved in the regulation of metabolism and physiological activities. Among the various microbial communities in intestinal tract, the bacterial community is of utmost importance (Pascale et al., 2018). Imbalance of intestinal microflora, or intestinal microecological dysbiosis, refers to disruptions in the composition, activity or distribution of microorganisms within the intestines. Such ecological disturbances or alterations in normal intestinal flora will affect intestinal permeability, intestinal mucosal barrier integrity and normal intestinal peristalsis, consequently resulting in a series of diseases (Canakis et al., 2020).

#### 3.4.2 The relationship between intestinal microbiome and NAFLD and related mechanism

Changes in intestinal microflora composition can alleviate or aggravate NAFLD through a variety of mechanisms. The main mechanisms include affecting fat production, modulating dietary energy metabolism, impacting related gene expression in the cholic acid metabolism signal pathway, and altering intestinal permeability. However, further research is required to fully understand the relationship between these factors and the development or progress of NAFLD (Safari and Gérard, 2019).

In terms of dietary energy metabolism, researches by Bäckhed F et al. have shown that germ-free mice, compare to those with intestinal microbiota, are resistant to high-fat or high-sugar diet-induced obesity. Intestinal bacteria produce secretory bacterial enzymes that facilitate the breakdown and digestion of polysaccharides in food, enhancing the absorption of food nutrients (Bäckhed et al., 2004; Bäckhed et al., 2007).

Alterations in intestinal flora can impact the integrity of the intestinal mucosal barrier including the structure of intestinal mucous layer, antimicrobial peptides and tight junction proteins, leading to increased intestinal permeability. This association is closely linked to the severity, occurrence and progression of NAFLD (Giorgio et al., 2014).

Bile acid can not only promote fat absorption, but also play the role of signal molecules in self-metabolism (Jiang et al., 2015; Safari and Gérard, 2019). In terms of bile acid composition changes, intestinal flora changes can increase bile acid metabolites and reduce liver triglyceride accumulation by inhibiting intestinal farnesol X receptor (FXR) signal. These effects are primarily achieved through the down-regulation of liver sterol regulatory element-binding protein 1C (SREBP1C) and decrease of *de novo* synthesis of fat. Inhibition of intestinal FXR/ceramide axis can mediate the development of NAFLD related with intestinal microbiota (Jiang et al., 2015).

#### 3.4.3 Potential probiotics in the treatment of NAFLD based on regulation of intestinal microbiota and its mechanism

In the study of Hu et al., *F. prausnitzii* strains (A2-165, LB8, ZF21, PL45 and LC49) could alleviate the symptoms related to glucose tolerance, liver steatosis, intestinal inflammation and oxidative stress in the NAFLD model (Hu et al., 2022). Notably, strains LC49 and LB8 were found to increase the production of short-chain fatty acids (SCFA) and regulate the composition of intestinal flora. The core microflora related to NAFLD include *Odoribacter, Roseburia, Erysipelatoclostridium, Tyzzerella, Faecalibaculum, Blautia* and *Acetatifactor.* Among them, the effects of *Erysipelatoclostridium, Tyzzerella, Faecalibaculum, Blautia* and *Acetatifactor* on the progress of NAFLD could be reversed by *F.prausnitzii* LC49 and LB8.

Patients with NAFLD exhibited a significantly lower total bacterial load compared to normal subjects, accompanied by a reduction in the abundance of various normal bacteria in the intestinal wall, including *Bifidobacterium, Lactobacillus, Lactococcus, True bacillus* and *Propionibacterium* (Vakhrushev et al., 2022).

*F.prausnitzii* LC49 and LB8 can enrich the abundance of *Lactobacillus, Enterobacter ileum, Bacillus faecalis, Duboxi* and *Bifidobacterium*, thus positively influences the metabolism of carbohydrates, amino acids and fatty acids. Moreover, *F.prausnitzii* LC49 and LB8 show significant anti-NAFLD effects and possess microbial regulatory properties, suggesting their potential as probiotic agents for the treatment of NAFLD (Hu et al., 2022; Shu et al., 2025).

## 4 Conclusions

While significant progress has been made in understanding the therapeutic potential of probiotics for NAFLD, several limitations persist in the current body of research. First, many studies lack long-term follow-up data, which is crucial to determine the sustainability of probiotic effects. Additionally, variations in probiotic strains and dosages across studies make it difficult to draw definitive conclusions about their optimal use. Most clinical trials have small sample sizes, limiting their generalizability. Furthermore, the mechanisms through which probiotics exert their therapeutic effects in NAFLD are not fully understood, with many studies relying on indirect markers of disease progression.

Despite promising therapeutic effects, challenges remain in probiotic application for MAFLD, such as the strain-specific nature of probiotics, host genetic and microbiota variability, and concerns regarding long-term safety and regulatory standardization. These issues underscore the need for rigorous clinical trials and mechanistic studies to validate efficacy and safety across diverse populations.

**Figure 1:**
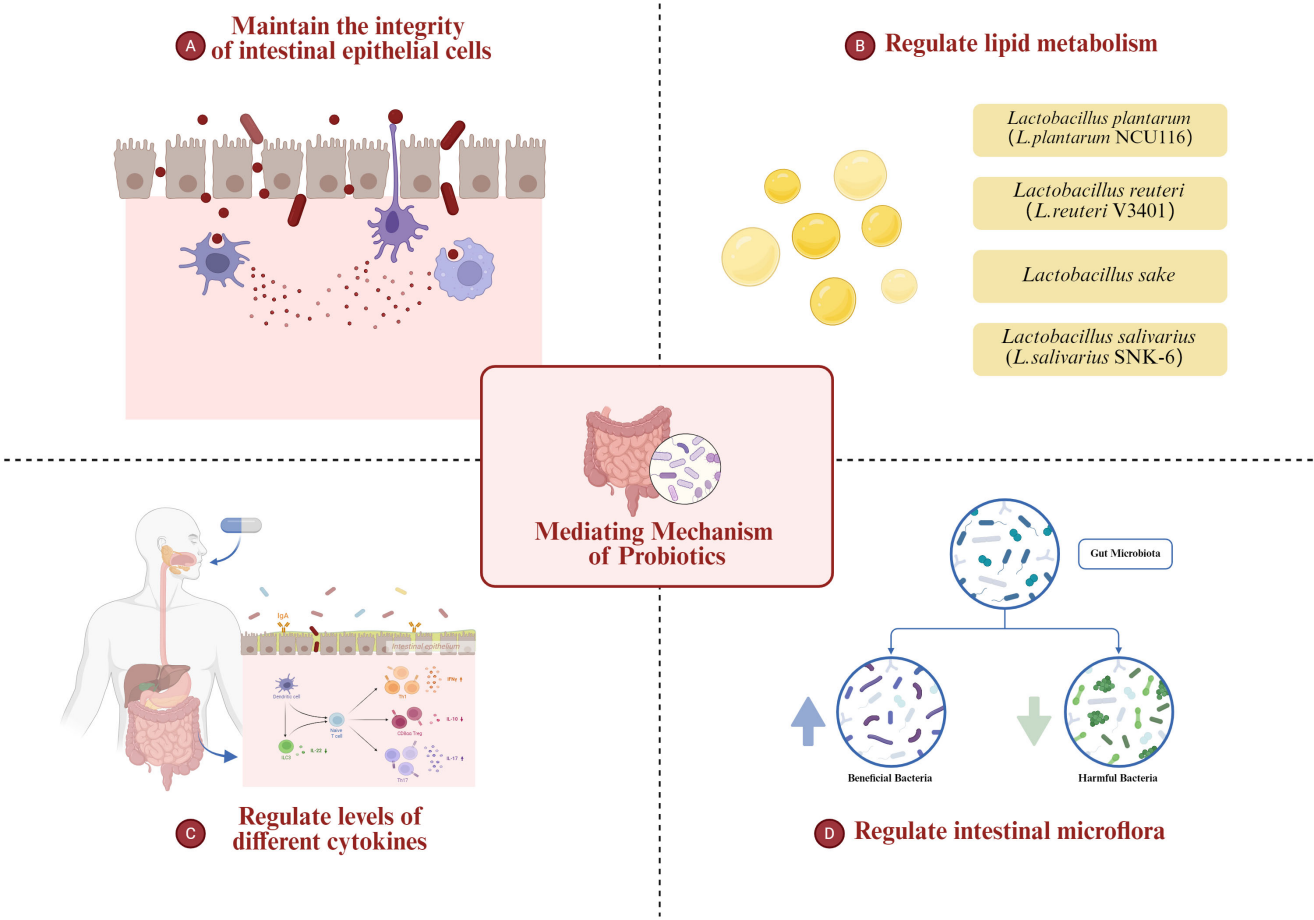
The role of probiotics in regulating the gut-liver axis in MAFLD, including modulation of intestinal microbiota, improvement of intestinal barrier integrity, alteration of lipid metabolism, and attenuation of inflammatory pathways.

Future research should focus on large-scale, multicenter trials with longer follow-up periods. A more standardized approach to the selection and dosage of probiotic strains is essential for comparing results across studies. Furthermore, research into the specific molecular mechanisms by which probiotics modulate gut microbiota and influence liver function is crucial for developing targeted therapies. There is also a need for studies that explore the combined effects of probiotics with other therapeutic interventions, such as dietary modifications or pharmaceutical agents. Addressing these challenges will provide clearer insights into the role of probiotics in managing NAFLD (**Figure 1**).
